# Comment on: Multifocal papillary thyroid microcarcinomas: is the total tumor diameter associated with the tumor behavior? A retrospective cohort study

**DOI:** 10.31744/einstein_journal/2026CE2169

**Published:** 2026-01-28

**Authors:** Ilker Sengul, Demet Sengul

**Affiliations:** 1 Giresun University Faculty of Medicine Thyroidology Unit Giresun Turkey Thyroidology Unit, Giresun University Faculty of Medicine, Giresun, Turkey.; 2 Giresun University Faculty of Medicine Division of Endocrine Surgery Giresun Turkey Division of Endocrine Surgery, Giresun University Faculty of Medicine, Giresun, Turkey.; 3 Giresun University Faculty of Medicine Department of General Surgery Giresun Turkey Department of General Surgery, Giresun University Faculty of Medicine, Giresun, Turkey.; 4 Giresun University Faculty of Medicine Department of Pathology Giresun Turkey Department of Pathology, Giresun University Faculty of Medicine, Giresun,, Turkey.

Dear Editor,

We write in reference to the recently conducted article, ‘Multifocal Papillary Thyroid Microcarcinomas: is the Total Tumor Diameter Associated with Tumor Behavior? A Retrospective Cohort Study," by Nardi et al.^(1)^ While the authors’ efforts are commendable, we feel it is necessary to critically assess some of their findings and limitations. The primary conclusion of the study suggests that total tumor diameter (TTD)—defined as the sum of the largest diameters of all tumor foci—shows no significant association with initial tumor aggressiveness or the patient's response to therapy after one year in cases of multifocal Papillary Thyroid Microcarcinomas (PTmC). This conclusion warrants further consideration, as the authors acknowledge that the prognostic significance of TTD remains debated, with previous research indicating that TTD greater than 10mm^(2,3)^ may be associated with a more aggressive prognosis, including increased risks of capsular invasion and central lymph node metastasis. However, it is noted that neither TTD nor the largest tumor diameter (a factor often associated with tumor aggressiveness) showed an association with AJCC staging or the risk of recurrence within this specific cohort. One must consider the limitations inherent in the methodology used in this study. The research, a retrospective cohort analysis utilizing convenience sampling, included 52 cases of multifocal PTmC. Given the small sample size, caution is warranted in drawing broad conclusions regarding the prognostic insignificance of the TTD parameter. Additionally, the authors note that comparison with previous studies was challenging, as this investigation focused exclusively on multifocal PTmC, while others included unifocal cases. Despite these limitations, the study does present a significant finding, deserving of recognition: the total number of metastatic lymph nodes remained the sole independent predictor of AJCC staging. This is a noteworthy distinction, especially since the AJCC criteria themselves account for the presence of lymph node metastasis, yet do not explicitly account for the sheer number of affected nodes. The authors’ commitment to studying a uniformly treated group with strictly defined multifocal PTmC is commendable. However, the lack of an association between TTD and prognostic factors, which contrasts sharply with other studies in the literature, highlights the need for further robust investigations—preferably prospective studies with a larger patient cohort—to fully validate these findings. While the conclusion that TTD has limited prognostic value is a key takeaway, the real insight provided by this study lies in its demonstration of the critical prognostic importance of the number of metastatic lymph nodes. Future prospective studies building on these findings are essential to refine personalized treatment strategies and supportive care approaches. This issue warrants further exploration. We thank Nardi et al.^(1)^ for their important research^(4)^ conducted in São Paulo, which will contribute significantly to the field of thyroidology.

## Data Availability

The underlying content is contained within the manuscript.
